# The role of tumor-associated macrophages in primary hepatocellular carcinoma and its related targeting therapy

**DOI:** 10.7150/ijms.56003

**Published:** 2021-03-15

**Authors:** Lu Deng, Kang He, Yixiao Pan, Hai Wang, Yi Luo, Qiang Xia

**Affiliations:** Department of Liver Surgery, Renji Hospital, School of Medicine, Shanghai Jiao Tong University, Shanghai, China.

**Keywords:** Tumor-associated macrophages, hepatocellular carcinoma, therapy

## Abstract

Liver macrophages consist of ontogenically distinct populations termed Kupffer cells and monocyte-derived macrophages. Tumor-associated macrophages (TAMs) inhepatocellularcarcinoma (HCC) play a prominent role in tumormicroenvironment by presenting M1(induced by IFN γ along with LPS) and M2(induced by IL-4 and IL13) polarization. Although TAMs are involved in tumor immune surveillance during the course of HCC, they contribute to tumour progression at different levels by inhibiting the anti-tumor immune response, promoting the generation of blood vessels and lymphatic vessels, and supporting the proliferation and survival of tumor cells. In this paper, the multiple functions of TAMs in HCC were reviewed to provide assistance for future researches about therapeutic approaches.

## Introduction

HCC accounts for 75-85% of primary liver cancer, making it the sixth most common cancer in the world and the fourth leading cause of cancer-related death 1. Macrophages, as the main component of leukocyte infiltration, play an important role in different types of tumor-related inflammation and are closely related to the outcome of treatment. Analyzingfunctions ofTAMsin the process ofprimary HCC can not only help with treatment of HCC, but also promote researches about functions of other inflammatory cells and factors in tumor-related inflammation. Herein, we provide a brief overview of the role of TAMs in the development and progression of HCC, and review the current emerging macrophage targeting therapies associated with TAMs.

## 1. Classification and polarization of TAMs in HCC

Macrophages are particularly rich in the liver, and mainly divide into liver-resident macrophages, termed Kupffer cells, and blood/bone marrow-derived macrophages, termed monocyte-derived macrophages. Under the condition of liver injury leading to the reduction of Kupffer cells or experimental removal of Kupffer cells, monocyte-derived macrophage can replace Kupffer cells 2, 3. Monocyte-derived macrophages can then acquire a phenotype that is virtually indistinguishable from Kupffer cells 4.

TAM is an important part in tumor associated inflammation and can be polarized to disparate functional phenotypes, as M1 (induced by IFN γ along with LPS) and M2 (induced by IL-4 and IL13) macrophages 5. IL-10, corticosteroids, andmolecules and immune complexes released by apoptotic cells canalsoinfluence functions of monocyte-derived macrophages.These signals induce macrophages to express some of the same functional phenotypes as M2 cells (such as expression of scavenger receptors), but these cells are different from M2 cells (such as chemokines). This group of cells is termed M2 like 6. Present studies do not distinguish between M2-TAMs and M2-like TAMs, nor do M1-TAMs and M1-like TAMs.Studies have shown that the more infiltration of M2-TAMs, the worse its clinical prognosis will be by inducing tumor cell proliferation, angiogenesis, tumor metastasis and epithelial-mesenchymal transition (EMT) 7-9. Though it has reported that CD68+HLA-DR + M1-like TAMs may also enhance motility of HCC cells via NF-κB/FAK pathway 10, it is generally believed that M2 TAMs play pro-tumor role while M1 TAMs exhibit anti-tumor functions 11. Kupffer cells are considered as specific TAMs in HCC microenvironment. Kupffer cells and monocytes-derived macrophages are both highly plastic. They can adjust their functional phenotypes according to signals from HCC microenvironment 12, 13. Most researches on macrophage polarization are simply implemented *in vitro*, which neglect the tumor microenvironment *in vivo* and rarely distinguish Kupffer cells from monocyte-derived macrophages. Thus, the polarization and activation of TAMs may involve more stimuli agents and more complex mechanisms *in vivo* 14.

## 2. TAMs in HCC surveillance

Cellular senescence is a stress response mechanism that induces cells at risk of malignant transformation to stop proliferating, and is therefore widely recognized as an anti-tumor mechanism. Senescent cells can also secrete specific cytokines, including IL-1 and IL-6, known as senescence-associated secretory phenotype (SASP). These cytokines can affect adjacent tissues and immune cells 15. Because specific SASP can induce changes of chromatin, the cell cycle of most senescent hepatocytes stop 16. In liver, cellular senescence mechanism is associated with inhibition of liver cancer progression 17, 18. Under the stimulation of oncogenic stress, the activation of the p53/p21 and p16/Rb anti-tumor pathways is an important trigger factor of senescence. These pathways promote cellular senescence by activating genes that inhibit cell cycle progression and promote senescence 19-22. If additional mutations of the proto-oncogene P53 are induced, resulting in the cessation of cellular senescence, liver cancer will develop progressively 23, 24.

Cells that have undergone oncogenic transformation, precancerous liver cells and senescent liver cells coexist in cancerous liver 25. Senescent hepatocytes, especially senescent precancerous cells, produce many chemokines, among which C-C motif chemokine 2 (CCL2) can recruit NK cells to achieve the clearance of tumor cells 23, 24. CCL2 can also recruit lots of monocyte-derived macrophages, likeC-C chemokine receptortype 2(CCR2)+ macrophages. CCR2+ macrophages and T cells work together to clear senescent liver cells and prevent the progression of liver cancer 26. However, in cells that have progressed to HCC, recruitment of CCR2+ macrophages leads to aggregation of M2-TAMs or myeloid-derived suppressor cells(MDSCs) benefiting tumor growth. M2-TAMs and MDSCs inhibit CD8 T cells and NK cells, thus promoting the growth of HCC and worsening the prognosis and survival of HCC patients 26, 27.

## 3. TAMs promoting HCC progress

### 3.1. TAM promotes angiogenesis and lymphogenesis

Tumor angiogenesis provides essential nutrients and oxygen to tumor tissues, which is one of the basic requirements for tumor growth and proliferation, and also provides vascular pathways for tumor metastasis. Some clinical studies have shown that the degree of vascular distribution in HCC tissues is related to the severity of the disease 28. TAMs can stimulate angiogenesis by secreting angiogenic factors, or indirectly angiogenesis by secreting angiogenic factors, or indirectly by producing extracellular matrix-degrading proteases, which in turn release sequestered angiogenic factors 29. Theseangiogenesis factors can stimulate chemotaxis ofendothelial cells and macrophages 30, 31, one of which is Tyrosine kinase with Ig and EGF homology domains 2(TIE2). TIE2 receptor expressing monocytes (TEMs) increase a lot in peripheral blood and the liver of HCC patients, which is of diagnostic value for HCC. In addition, the density of TEMs in HCC tissues is related to the degree of angiogenesis. Therefore, TEMs may represent a new cell marker for the diagnosis of HCC that reflects the degree of hepatic angiogenesis 31.

A majority of studies mainly explored the interaction between HCC cells and TAMs under normoxic conditions, whereas in patients, HCC tumor mass is exposed to varying degrees of hypoxia. Inflammation and hypoxia can reinforce each other in tumor microenvironment. On one hand, hypoxia enhances the proinflammatory activity of innate immune cells such as neutrophils and macrophage 32. On the other hand, oxygen consumption of innate immune cells increases in response to the sudden burst of cytokine release. At the same time, NF-κB and Hypoxia Inducing Factor-1(HIF-1) are also mutually activated 33.

The distribution and function of TAMs are sensitive to hypoxia-related stress. For example, TAMs tend to cluster in the hypoxic areas of the tumor 34 and have the stronger ability to promote angiogenesis under hypoxia 35. HIF-1 is also stable under hypoxia 36.It was found that TAMs secreted more Interleukin-1β (IL-1β) due to increased stability of HIF-1α in moderate hypoxia. Under persistent and severe hypoxia, the necrotic debris of HCC cells induced potent IL-1β release by TAMs with an M2 phenotype through Toll-like receptor 4(TLR4)/TIR domain-containing adapter-inducing interferon-β (TRIF)/NF-κB signaling pathway. Following the increase of IL-1β in the local microenvironment, the synthesis of HIF-1α was up-regulated by IL-1β in HCC cells through cyclooxygenase-2. Overexpression of HIF‐1α enhances EMT of HCC cells and IL‐1β promotes HCC metastasis 8.

TAM promotes lymphangiogenesis mainly through two mechanisms. One is secretion of lymphangiogenic factors, suchas vascular endothelial growth factor-C and -D(VEGF-C and VEGF-D) 37. Angiogenic factors, vascular endothelial growth factor-A (VEGF-A) and matrix metalloproteinase 9(MMP9), secreted by macrophages also promote lymphangiogenesis 38, 39. The other is promoting tumor lymphangiogenesis through trans-differentiation to lymphatic endothelial cells. TAM can express LYVE-1, one of the markers of lymphatic endothelial cells 40, but there are still some doubts about this theory 41. However, the mechanism of TAMspromotinglymphangiogenesis has not been confirmed in HCC.

### 3.2. TAMs andsuppression of antitumoral immune responses

#### 3.2.1. Factors inducing M2 polarization of TAMs

Current studies have shown that IFN-γ and LPS induce TAMs to polarize toward M1, which mediates Th1 response, and are associated with inducing death of tumor cells. In contrast, M2-TAMs, which mediate Th2 response, is polarized by IL-4 and IL-13 and has an immunosuppressive effect, promoting tumor progression 5, 9, 11. The role of IL-10 in TAM polarization is still in question. Some studies have shown that IL-10 can induce TAMs to transform into the M2 phenotype 42, but there are also studies suggesting that it just plays a supporting role 43.

Oxidored Nitro Domain Containing Protein 1(NOR1) can overexpress in human HCC tissues and is associated with poor prognosis. However, the overexpression of NOR1 protein in human HCC cell lines has no effect on cell proliferation and migration. The poor prognosis of HCC is mainly related to the overexpression of NOR1 protein in CD163+ M2-TAMs. The reason is that overexpression of NOR1 gene in M2-TAMs promotes the development of N-nitrosodiethylamine (DEN) induced HCC by promoting the M2-selective polarization of TAMs 44.

In addition, the inflammatory cytokines produced by TAMs can promote the differentiation and amplification of IL-17 producing T cells 45, 46. After direct interaction with IL-17 + cells at the edge of tumor invasion, tumor cells acquired the ability to induce CXCR3+ B cell recruitment and maturation. CXCR3+ B cells can induce M2b polarization ofmacrophages through IgG-dependent pathway, thus creating conditions for tumor growth 47.

Treatment with CSF-1R inhibitor PLX3397 delayed tumor growth in both xenograft and allograft models, and this delay was probably mediated by the transition from M2 macrophages to M1 macrophages in a TAM population induced by blockade of the CSF1/CSF-1R signal pathway 48.

#### 3.2.2. Mechanisms of M2-TAMssuppressing antitumoral immune responses

##### 3.2.2.1. Relationships between TAMs and T cells

T cells and NK cells play a key role in the elimination of tumor cells and are closely related to the polarization of TAMs. Overall, TAMs infiltration is proportional to CD4+CD25 + FoxP3 T cells (Tregs) and inversely proportional to CD8+T cells (cytotoxic T cells) in the HCC environment. Blocking CSF1R on TAMs will increase the infiltration of CD8+ T cells and decrease the infiltration of CD4+ T cells 48. Cantharidin can induce a decrease in CD4+CD25 + FoxP3 T cells by promoting the polarization of TAMs towards M2 49. Increased infiltration of Tregs (CCR4+) may be due to TAMsincreasing secretion of CCL17. In turn, CD69+T cells release IFN, which will cause TAMs to produce inhibitor 1-methyl-DL-tryptophan (IDO) and lead to tumor promoting function 50. IDO protein is a tryptophan degrading enzyme, which has a strong control on T cell response. Experiments *in vitro* show that suppressive macrophages contacting autologous T cells can restore the ability to produce IL-12, activate T cells and produce IFN-γ. IFN-γ, in turn, lead macrophages to express IDO, and finally suppress anti-tumor functions of T-cells. TAMs can also produce immunosuppressive cytokines (such as IL-10 and TGFβ) that affect the proliferation and differentiation of T cells, and also produce CCL5, CCL20, and CCL22 to recruit nTreg cells. The reduction of T cells may be related to the FASL/FAS pathway 51.

##### 3.2.2.2. Relationships between TAMs and NK cells

In patients with advanced HCC, the number of NK cells decreases significantly, accompanied by decreased production of TNF-α and IFN-γ. The high infiltration of TAMs is positively correlated with impaired functional activity of NK cells in HCC tissues. CD48 protein is highly expressed in TAMs in HCC tissues, which binds to the 2B4 receptor on NK cells, leading to rapid activation of NK cells, followed by failure and death 52. Sorafenib can mediate the activation of NK cells, enhance the cytotoxicity of NK cells to tumor cells and promote anti-tumor function of NK cells. However, TAMs are crucial for sorafenib activating NK cells. Blocking experiments confirmed the presence of cytokine signaling between two types of cells, which is related to cell contact. Non-secreted surface cytokines on the surface of macrophages may explain the activation of NK cells related to cell contact 53.

##### 3.2.2.3. Other immunosuppressive reactions related to TAMs in HCC

Current studies have reported that the inhibition of Kupffer cells on CD8+ T cells in HCC is mediated by PD-1/PD-L1 interaction 54. However, the overexpression of PD-L1 in HCC is also associated with TAMs 55. TAMs also express PD-1, which is negatively correlated with the ability to engulf tumors. Blocking PD-1/PD-L1 signaling pathway *in vivo* can improve the phagocytosis of macrophages, reduce tumor growth and prolong the survival time of mice 56. IL-17 secreted by monocytes or macrophages may also inhibit T cell function by promoting the expression of PD-L1 in HCC 55.

Major histocompatibility complex class I(MHC class I) also plays an important role in the control of macrophage reactions. Tumor cells express common MHC class I component β2-microglobulin (β2M), and TAMs up-regulate expression of suppressive receptor, leukocyte immunoglobulin like receptor B1 (LILRB1), on the cell surface. This signaling pathway can directly block phagocytosis of tumor cells. *In vivo* or *in vitro* experiments, blocking MHC class I or LILRB1 activates phagocytosis of tumor cells, indicating the importance of this signaling pathway in suppressing anti-tumor immune response 57.

### 3.3. TAMspromote proliferation and survivalof tumor cells

#### 3.3.1. The pathways that M2-TAMs promote transformation of HCC cells into CSCs

Cancer stem cells or cancer-initiating cells (CSCs) are a kind of tumor cells with self-renewal ability and pluripotency. Many studies believe that CSCs with multiple characteristics of stem cells may be the reason for tumor recurrence after treatment, development and metastasis 58, 59. CSCs may not be a fixed group of cells, and have plasticity subject to the regulation of tumor microenvironmental factors. Many cell surface markers have been identified to define human HCC CSCS, including CD24, CD44, CD90, CD117, CD133, and epithelial cell adhesion molecule (EpCAM), of which CD44 is an important marker 60-62.

It has shown that M2-TAMs promoted CSCs amplification and tumorigenesis through the IL-6/STAT3 signaling pathway 63, which can transform HCC cells into CSCs from the perspective of cell surface markers. M2-TAMs promote migration and EMT of HCC cells through TLR4/STAT3 signaling pathway 64. Other hormones and growth factors also participate in this pathway by activating Janus kinase (JAK), and then, downstream STAT3 is phosphorylated to form dimers that mediate nuclear translocation and DNA binding associated with cell proliferation and survival. TAMs can also promote HCC cells to gain CSC-like features through EMT mediated by TGF-β1 65, 66.

#### 3.3.2. Other pathways of TAMs promoting proliferation and survival of tumor cells

TAMs can lead to deterioration of HCC by producing other proteins. Allograft inflammatory factor-1 (AIF-1) is a cytoskeletal related protein that can increase proliferation and migration of macrophages 67. CSF1 induces the expression of AIF1 in macrophages by activating the CSF1R-MEK1/2-Erk1/2-c-Jun signaling pathway 68.It was found that CSF1 can also induce activation of CSF1R-PI3K-PLCγ signaling pathway. AIF1, together with phospholipase Cγ (PLCγ), further activates RAC signaling pathway related to cytoskeleton rearrangement, leading to proliferation and migration of macrophages 69. The expression of AIF1 in TAMs also increases proliferation and migration of HCC cells *in vitro*, and promotes growth of tumor in animal models 68. At the same time, AIF1 over-expressed macrophages are accompanied by increased secretion of CXCL16, which is also one of the mechanisms by which AIF1 promotes migration and infiltration of HCC 70.

A study has demonstrated that secretion of M2 macrophage-derived CCL22 in HCC could be induced by tumor cells, and in return that CCL22 could facilitate HCC cells during migration and dissemination via EMT activation 9. The transcript level of CCL22 was significantly over-expressed in the peritumoral but not in the intratumoral region as well as in normal liver tissues, while expression of the corresponding receptor CCR4 was higher in most HCC cell lines as well as in clinical tumoral tissues compared with hepatocytes and peritumoral tissues. M2 macrophage-derived CCL22 was proved to be a potent chemoattractant for HCC cells and the CCL22-CCR4 mediated migration was inhibited in MHCC97L cells treated with the CCR4 antagonist C-021. Immunoblot analysis revealed that the induction of EMT was through Smad2/3 and Smad1/5/8 activation and Snail upregulation in MHCC97L cells. Based on such evidence, it can be speculated that HCC cells could possibly employ CCR4 to migrate from the bulk tumor towards peritumoral regions where CCL22-producing M2 macrophages predominantly reside.

CXCL8 is one of chemokines of proinflammatory CXC (C-X-C Motif) 71. Macrophages activated by co-cultured HCC cells can produce high levels of CXCL8. Under stimulation of CXCL8, expression of miR-18a and miR-19a (belonging to miR-17 group) increases in HCC and leads to enhanced proliferation and metastasis of HCC cells 72.Besides, as previously mentioned, CCL2/CCR2 axis promotes the development of HCC in cells that have advanced to tumor cells.

## 4. Treatment targeting macrophages

### 4.1. Treatment targeting suppressing functions of M2-TAMs

A number of drugs targeting inhibiting functions of M2-TAMs have been approved for clinical trials against different types of cancer, such as the CSF‐1R inhibitor and antibody for the treatment of diffuse giant cell tumor of the tendon sheath 73, CCL2 antibody and antagonist for treatment of metastatic prostate cancer and so on^74^. However, in the field of HCC, apart from researches on TAMs by using sorafenib, no other drugs related to the inhibition of M2-TAMs have been put into clinical trials.

Combretastatin a-1 phosphate (CA1P) is a microtubule polymerization inhibitor that binds to colchicine binding sites of microtubuloproteins. In addition to inducing apoptosis of HCC cells, CA1P shows anti-TAMs activity in mice. *In vitro* studies have shown that CA1P induces microtubule depolymerization-mediated AKT inactivation, which resulted in GSK-3β activation, Wnt/β-Catenin pathway inhibition, and Mcl-1 down-regulation. The Wnt/β-Catenin signaling pathway is reported to regulate the polarization of macrophages into M2, while the down-regulation of Mcl-1 will cause the accumulation of ROS and lead to cell apoptosis. Experiments invivohave demonstrated that CA1P reduces TAMs in the tumor microenvironment and reduces the expression of TGF-β and TNF-α 75.

As previously mentioned, the level of IL6 in human HCC samples is related to tumor stage and CSCs level. Upregulating receptor-interacting protein 140 (RIP140) can inhibit NF-κB/IL-6 axis in TAMs to suppress HCC 76. Tocilizumab, a drug for treatment of rheumatoid arthritis approved by FDA, can block IL6 signaling and inhibit CSCs activity stimulated by TAMs. It was suggested that this drug may be effective in the treatment of HCC 63.

### 4.2. Modulating TAMs to assist other anti-tumor drugs

Sorafenib is a multikinase inhibitor that has been widely used in the treatment of HCC. Currently, sorafenib is one of the first-line systematic standard treatment for patients with advanced HCC, and the patient's average survival benefit is 3 months 77. Sorafenib is not that effective, which may be related to infiltration of TAMs 78. Sorafenib-induced hypoxia may increase the levels of CSF1, HIF, and CCR4, thereby affecting TAM invasion and leading to tumor progression 8, 68, 79. As previously mentioned, the infiltration of macrophages leads to the overexpression of PD-L1 on the surface of HCC cells, which may also be one of the reasons for sorafenib resistance 55.It was found that a natural product from Abies georgei, structurally related to kaempferol, can be used as a CCR2 antagonist. In mice model with HCC, inhibiting the recruitment of TAMs by inhibiting CCR2 can enhance the efficacy of sorafenib 80. Compared with Sorafenib, CA1P is likely to be more effective while reducing drug resistance 75.

Zoledronic acid (ZA) can inhibit bone resorption and is mainly used to treat pain caused by bone metastasis of metastatic tumors 81. Some scholars applied ZA in liver tumors and found that ZA, as TAM inhibitor, improved the anti-tumor effect of Sorafenib 82. Other studies have shown that combined treatment with ZA can enhance the inhibitory effect of hepatic transcatheter arterial chemoembolization (TACE) on HCC tumor growth, in which ZA had a significant inhibitory effect on the infiltration of TAM after TACE. In addition, ZA can also inhibit the secretion of VEGF after TACE 83.

Although the application of CAR-T in primary HCC has been rarely reported, it has been confirmed that partial fusion of CAR-T can delay tumor progression in mice with metastatic liver cancer, but immunosuppression in liver regulated by MDSC prevents complete tumor clearance. Therefore, further study of CAR-T and TAMs combined target therapy may solve this problem 84.

### 4.3. Immune checkpoint therapy

Nivolumab is an IgG4 antibody that causes immune checkpoint blockade by diminishing inhibitory signaling through the programmed death receptor-1 pathway 85. Based on the encouraging results in Phase I/II trials, nivolumab was approved by the FDA for liver cancer as a second line treatment after failure of sorafenib. Anti-PD-1 therapy can enhance the anti-tumor immune response of HCC models. However, this therapy only shows efficacy with targeted drugs aiming hypoxia like CCR4 inhibitors under sorafenib treatment background 79. As previously mentioned, the elimination of TAMs provides a new approach for dealing with HCC afterPD-1 therapy tolerance. The relationship between the expression level of PD-1/PD-L1 on TAM and Nivolumab responsiveness remains to be elucidated and carefully assessed 86, 87.

Lymphocyte-activation gene 3(LAG3) is also an important immune checkpoint. Fibrinogen-like protein 1(FGL1), a liver-secreted protein, is a major immunosuppression ligand of LAG3. FGL1 inhibits antigen-specific T cell activation, and ablation of FGL1 in mice promotes T cell immunity. FGL1 is overexpressed in tumor cells, and knockout of FGL1 can reduce the density of TAMs 88.

## 5. Conclusions

TAM inHCC has dual function owning to their different polarization. On the one hand, TAM is involved in tumor immune surveillance during the course of HCC. On the other hand, TAMs can promote the progression of HCC by inhibiting their anti-tumor immune response, promoting the generation of blood vessels and lymphatic vessels, and promoting the proliferation and survival of tumor cells. TAMs targeting therapy focuses on inhibiting tumor-promoting functions of M2 cells, modulating TAMs to assist other anti-tumor drugs and immune checkpoint therapy. TAMs may be an important potential target for the treatment of HCC due to its impact on the development of HCC and subsequent chemotherapy.

Plasticity and flexibility are key characteristics of mononuclear phagocytes and their activation states 77. Since macrophages are susceptible to the microenvironment, the polarized M1 or M2 phenotypes of macrophages can to some extent reverse to each other *in vivo* or *in vitro* 89. Macrophages that have induced polarization *in vitro* may not remain polarized after being transferred to the body. All these bring difficulties for TAMs targeting therapy.

## Figures and Tables

**Figure 1:**
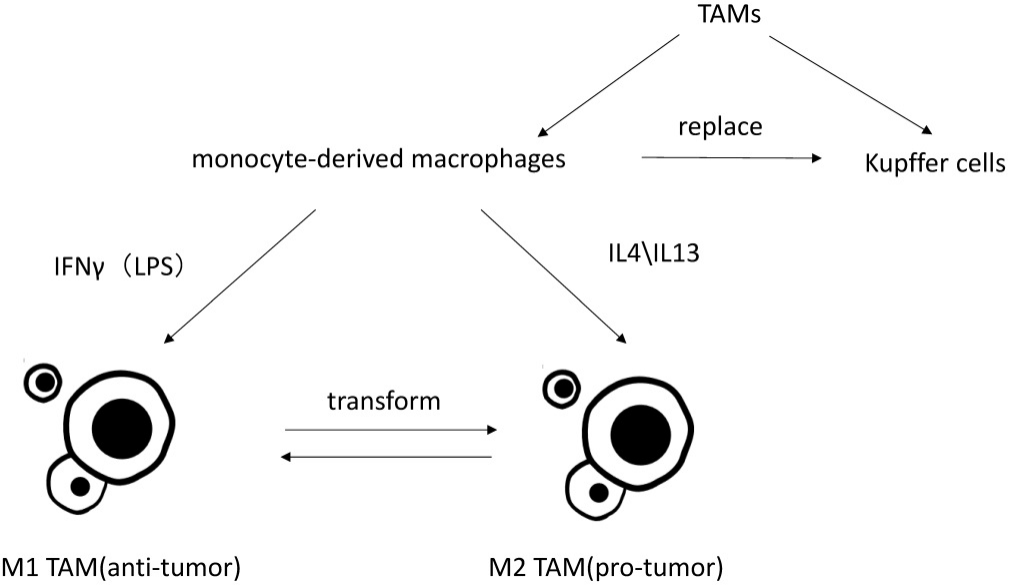
Classification of TAMs and differently polarization of TAMs *in vitro*.

**Figure 2:**
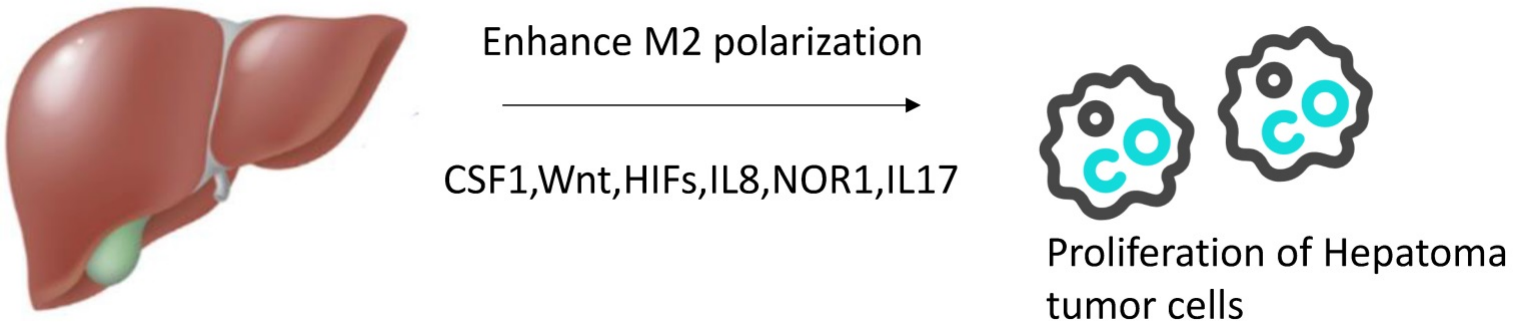
Agents enhancing M2 polarization of TAMs *in vivo*. In tumor microenvironment, more agents can promote TAMs transform to M2 phenotype such as CSF1,Wnt,HIFs,IL8,NOR1,IL17.
